# O04 Large vessel vasculitis related to COVID-19 infection

**DOI:** 10.1093/rap/rkaa053.003

**Published:** 2020-11-03

**Authors:** Zoe Rutter-Locher, Bhashkar Mukherjee, Thomas Mason, Begona Lopez

**Affiliations:** Guy's and St Thomas' NHS Trust, London, United Kingdom

## Abstract

**Case report - Introduction:**

By June 2020, 175,000 cases of COVID-19 had been identified in London alone. The most common symptoms include fever, headache, loss of smell, cough, myalgia, and sore throat. The major complication is acute respiratory distress syndrome (ARDS) but systemic complications such as cardiomyopathy, acute kidney injury, encephalopathy and coagulopathy are being identified. A delayed multi-system inflammatory syndrome in children has also been recognised and further complications are likely to be identified as our experience increases. Here, we report the case of a patient with large vessel vasculitis who initially presented with symptoms highly likely to be due to COVID-19 infection.

**Case report - Case description:**

A 36-year-old black African nurse presented in May 2020, with acute onset 7 days prior of high-grade fevers, rigors, nights sweats, generalised myalgia, sore throat, headache with photophobia, anosmia, dysgeusia and a widespread rash. She was a smoker with no other relevant medical, travel nor sexual history, and no drug use. A COVID-19 swab on day 2 had been negative and she had taken a course of Doxycycline.

Examination revealed firm palpable subcutaneous nodules on lower limbs, upper back and forehead and cervical lymphadenopathy. She was photophobic with no meningism. The rest of her physical examination was normal. BP was 116/97 mmHg, heart rate 109 bpm and satO2 100%. Investigations demonstrated C-reactive protein 330mg/L, erythrocyte sedimentation rate 140, Ferritin 479, lymphopaenia 0.7x10^9^, eGFR 54 with no haematoproteinuria, D-dimer 3.05 mg/L with INR 1.1, aPTT 1.3, fibrinogen 8.8 g/L. Hb, WCC, liver function, CK, serum ACE and triglycerides were normal. Infectious screen revealed negative blood cultures, HIV, Hepatitis B and C, EBV, CMV and Treponema pallidum serology. CT brain and CSF analysis were normal including bacterial culture and viral PCR. ANA, ENA, dsDNA, ANCA and aPL antibodies were negative with normal complement levels. Throat swab grew group A streptococcus and she was treated with broad spectrum antibiotics for 7 days maintaining fevers up to 39^o^C. Skin biopsy was non-specific with negative direct immunofluorescence but showed microvascular thrombi in the papillary dermis. COVID-19 PCR tests (three naso-pharyngeal swabs and one stool PCR) and IgG test (day 38) were negative. CT showed no pneumonitis but non-specific retroperitoneal stranding with medium/large vessel vasculitis involving both proximal renal arteries and a 6 cm segment of mid abdominal aorta on PET-CT. We started oral prednisolone 40mg with immediate resolution of her fevers, myalgia, and inflammatory markers, remaining well a month later.

**Case report - Discussion:**

Takayasu’s arteritis is the most common autoimmune large vessel vasculitis (LVV) affecting young females and involves inflammation of the arterial wall ultimately resulting in stenosis and obstruction of the vessel. However, it is rare in patients with African heritage and usually presents with a prolonged prodromal phase. Given the atypical presentation and symptoms consistent with COVID-19 infection we feel that this patients’ LVV may have been a complication of COVID-19 infection. The relationship between infections and vasculitis is complex. TB and syphilis cause aortitis and a relationship between infection and vasculitis has been proven in HBV associated PAN and HCV associated cryoglobulinemia.

Experimental data supports a possible association between CMV and herpes virus and Takayasu arteritis. It could, therefore, be hypothesised that COVID-19 infection can trigger LVV. Our patient had a throat swab positive for Streptococcus pyogenes which is an uncommon cause of infective endocarditis and mycotic aneurism, but this patient had no evidence of either endocarditis or aneurism formation and so it was felt the throat swab finding was incidental. Our patient had repeated negative COVID-19 nasopharyngeal swabs and a negative antibody test at day 38.

Although this argues against a diagnosis of COVID-19 related illness, the relative lack of information we currently have regarding sensitivities of the tests, at what point COVID-19 PCR becomes negative in the illness and when/if patients develop antibodies, means these negative tests in the presence of typical symptoms cannot exclude the diagnosis. We believe this case is extremely important to highlight a possible novel inflammatory complication of COVID-19 infection. We decided to treat this patient in line with guidance for the management of LVV, including the introduction of methotrexate, but it will be interesting to observe her long-term outcome.

**Case report - Key learning points**
 

- Increasing numbers of COVID-19 related systemic inflammatory conditions are likely to be recognised over the coming months. We present the case of patient with large vessel vasculitis who initially presented atypically with symptoms consistent with COVID-19 infection
- To identify these complications, COVID-19 symptoms questioning should be part of any routine medical history
- More information is required regarding the sensitivity of COVID-19 PCR and antibody tests to aid the diagnosis of these conditions
- The long-term management of inflammatory conditions associated with COVID-19 infection is not clear and a discussion is warranted as to whether DMARDs should be initiated

